# Association of peak expiratory flow with motoric cognitive risk syndrome among older adults

**DOI:** 10.3389/fnagi.2024.1412542

**Published:** 2024-08-07

**Authors:** Hui Xu, Xiangwen Gong, Kaiwang Cui, Xuerui Li, Long Chen, Yiyi Lu, Yangfang Liao, Jianping Liu

**Affiliations:** ^1^Big Data Center, Beijing Children’s Hospital, National Center for Children’s Health, Capital Medical University, Beijing, China; ^2^Department of Respiratory and Critical Care Medicine, Ganzhou Key Laboratory of Respiratory Diseases, Ganzhou Institute of Respiratory Diseases, The Fifth People’s Hospital of Ganzhou, Ganzhou, Jiangxi, China; ^3^Department of Geriatrics, Tianjin Medical University General Hospital, Tianjin Geriatrics Institute, Tianjin, China; ^4^School of Rehabilitation Medicine, Gannan Medical University, Ganzhou, Jiangxi, China

**Keywords:** motoric cognitive risk syndrome, peak expiratory flow, older adults, lung function, cohort study

## Abstract

**Background:**

The association between lung function and motoric cognitive risk syndrome (MCR) is unclear. We aimed to explore the association of peak expiratory flow (PEF) with MCR using cross-sectional and longitudinal analyses.

**Methods:**

Within the CHARLS, 5095 participants were included in the cross-sectional analysis, and 4340 MCR-free participants were included in the longitudinal analysis. The PEF was assessed with a lung peak flow meter. MCR was characterized by cognitive complaints and a slow walking speed with normal mobility and without dementia. Logistic regression, Cox regression, and Laplace regression models were employed for data analysis.

**Results:**

In this cross-sectional study, logistic regression analyses revealed that continuous PEF was associated with MCR (odds ratio [OR], 0.998; 95% confidence interval [CI], 0.998, 0.999), and the ORs (95% CIs) of MCR prevalence were 0.857 (0.693, 1.061) for the middle tertile and 0.665 (0.524, 0.845) for the highest tertile compared to the lowest tertile. In a longitudinal cohort study, continuous PEF was dose-dependently associated with the risk of MCR. Compared with those in the lowest tertile of PEF, the hazard ratios (95% CIs) of incident MCR were 0.827 (0.661, 1,036) for the middle tertile and 0.576 (0.432, 0.767) for the highest tertile. Furthermore, compared with the lowest tertile, the highest tertile was associated with a delayed onset time of MCR of 0.484 (95% CI: 0.151, 0.817) years.

**Conclusion:**

A higher PEF was related to a lower prevalence of MCR and a lower risk for MCR, and a higher PEF also prolonged the onset time of MCR.

## Introduction

The world’s population is aging in an increasingly critical situation due to increasing life expectancy and declining fertility, and the influence of dementia on health is anticipated to increase dramatically, placing a significant burden on social security (United Nations, 2020). Sensory, motor, and cognitive functions change in the normal aging process, with a significant decrease in walking speed and motor activity (Puig et al., 1988). Motoric cognitive risk syndrome (MCR) was first reported in 2013 as a novel indicator of predementia phase clinical syndrome by combining slow walking speed with subjective cognitive complaints (Verghese et al., 2013). Recent studies have demonstrated that a diagnosis of MCR can be used as a screening indicator for adverse health outcomes (Chhetri et al., 2017), including falls (Callisaya et al., 2016), disability (Doi et al., 2017), dementia, cardiovascular disease (Meiner et al., 2020), and mortality (Beauchet et al., 2019). As MCR can be assessed with a relatively less complex method, MCR could be considered a useful tool for comprehending the pathophysiological mechanisms involved in the long preclinical period that results in dementia. Hence, identifying protective or risk factors for MCR, particularly changeable factors, holds great significance in tackling the issues posed by cognitive function decline.

A range of lung function trajectories are present in health and disease and could have significant clinical consequences (Agusti and Faner, 2019). PEF, a quickly and easily administered measurement of lung function, is identified as the maximum flow produced by the maximum force from total lung volume (Beauchet et al., 2019). Thus, PEF is a practicable indicator of general robustness and is workable in large-scale surveys and clinical contexts (Trevisan et al., 2019).

However, the research investigating the direct association between PEF and MCR is relatively limited. Several explanations could contribute to this association: Firstly, PEF as a key measure of respiratory function, may influence the brain’s oxygen supply and cognitive function. Studies suggest a link between good respiratory health and enhanced cognitive abilities, possibly because sufficient oxygen supports brain neuron activity, boosting cognition (Ngwenya et al., 2016; Donahue et al., 2023). Secondly, PEF is tied to overall health and lifestyle choices. People with higher PEF tend to adopt healthier habits like regular exercise and balanced eating, which are known to benefit cognitive and motor functions (Cotman and Berchtold, 2002). Considering the rational explanation for the association between PEF and MCR and the scare direct study in present, to explore whether and to what extent PEF influenced MCR, we assessed the cross-sectional and longitudinal relationships of PEF with MCR in older Chinese adults.

## Materials and methods

### Study population

The study population was sourced from the China Health and Retirement Longitudinal Study (CHARLS), an ongoing longitudinal cohort of individuals ≥ 45 years of age. Previous studies have elucidated the study design and assessment protocol for CHARLS (Zhao et al., 2014). In summary, multistage probability sampling was used to recruit individuals from 450 communities and administrative villages spanning 28 provinces in China. Upon enrollment, every individual completed a standardized questionnaire and anthropometric measurements. Afterward, these individuals were followed every two years. The Biomedical Ethics Review Committee of Peking University approved the CHARLS (IRB00001052-11015). Each individual was allowed to share their data and signed a consent permitting disclosure. Data from the 2011, 2013, and 2015 CHARLS cohorts were collected for our study, and the data can be requested at http://charls.pku.edu.cn/.

**FIGURE 1:**
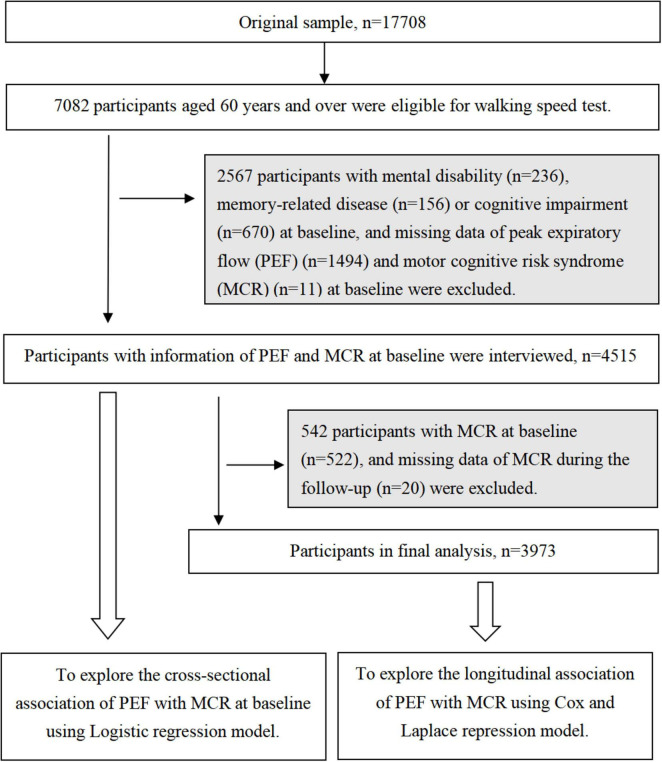
Flowchart of the study participants.

At baseline, 17708 participants aged ≥ 45 years were enrolled, with 7082 (39.9%) individuals aged ≥ 60 years meeting the criteria for the walking speed assessment. Among the 7082 individuals, we excluded 1987 participants with mental disabilities (*n* = 236), memory-related diseases (*n* = 156), and missing data on PEF (*n* = 1584) and MCR (*n* = 11) at baseline. Thus, 5095 participants were enrolled in this cross-sectional study. Furthermore, individuals with MCR at baseline (*n* = 600) and individuals with memory-related disease (*n* = 135) or missing MCR data (*n* = 20) during the follow-up period were excluded. Finally, 4,340 participants were enrolled in the longitudinal cohort study (Figure 1).

### Assessment of PEF

The PEF was measured and recorded by train technicians with a lung peak flow meter and a disposable mouthpiece. Participants in the stand-up activity were directed to breathe deeply, hold the mouthpiece, cover it completely with their mouth, avoid air leakage, and then blow into the mouthpiece as fast and hard as they could. PEF was assessed 3 times with an interval of 30 seconds for each time in units of L/min, and the maximum value of the 3 times was used in the present study. A higher PEF indicated better lung function.

### Assessment of MCR

MCR was characterized by cognitive complaints and a slow walking speed with normal mobility and without dementia, following a previously established protocol (Bai et al., 2021).

Cognitive complaints were assessed by asking participants to self-rate their current memory. Individuals who rated their memory as fair or poor were considered to have cognitive complaints, while others were considered free of cognitive complaints.

Slow walking speed was established as one standard deviation below the average walking speed for the age- and sex-specific population at baseline. All participants ≥ 60 years were eligible for the walking test and were categorized into groups for every 5-year age interval. However, participants with a disease (e.g., ankle sprain) that hindered their walking ability did not complete the walking speed test. Individuals were directed to walk at their normal speed on the walkway. Participants were allowed to use walking aids if necessary. The number of times the participants walked across the walkway twice was recorded by a stopwatch, and the average time was calculated. Walking speed was computed as the distance traveled by 2.5 meters divided by the average time in seconds (m/s). The slow walking speed in this study was defined as < 0.51 m/s in men aged 60–64 years, < 0.49 m/s in men aged 65–69 years, < 0.45 m/s in men aged 70–74 years, < 0.43 m/s in men aged 75–79 years, < 0.38 m/s in men aged ≥ 80 years, < 0.47 m/s in women aged 60–64 years, < 0.44 m/s in women aged 65–69 years, < 0.43 m/s in women aged 70–74 years, < 0.40 m/s in women aged 75–79 years, and < 0.36 m/s in women aged ≥ 80 years.

### Assessment of potential confounders

Information on age, sex, education, smoking status, alcohol consumption, and chronic illnesses (e.g., hypertension, diabetes) was obtained through a uniform structured questionnaire administered by a trained interviewer. Body mass index (BMI) was determined by dividing weight by height, with the unit expressed in kilograms/meters squared (kg/m^2^). Weight and height were evaluated using a height and weight scale with participants wearing lightweight clothing and no shoes by standard measurement procedures. Education was classified into three groups: illiterate, primary school and below, or middle school and above. Smoking status and alcohol consumption status were also classified into three groups: never, former, and current. All chronic illnesses were classified as yes or no.

### Statistical analysis

Differences in characteristics among individuals with or without MCR in the cross-sectional study and among individuals with the lowest, middle, and highest PEFs in the longitudinal study were tested by one-way analysis of variance or Kruskal-Wallis tests for continuous variables, while chi-square tests were used for categorical variables.

The odds ratios (ORs) with 95% confidence intervals (CIs) were calculated by logistic regression models to assess the cross-sectional association between PEF (continuous and tertiles) and MCR at baseline. The hazard ratios (HRs) with 95% CIs were calculated by a Cox regression model to assess the longitudinal association between PEF and MCR during follow-up. The proportional hazards assumption of the Cox regression model was not violated after testing. The 10th percentile differences (PDs) in the longitudinal relationship between the MCR onset time and the PEF were calculated via a Laplace regression model. The models were initially adjusted for age, sex, and education, and further additional adjustments were made for BMI, smoking status, alcohol consumption, and chronic diseases.

In the sensitivity analysis, the missing covariate data were imputed for 252 individuals in the cross-sectional or longitudinal study using multiple imputations via a chained equation. Furthermore, we conducted a repeated analysis of the models when we excluded 2278 participants with lung disease (*n* = 710) or arthritis (*n* = 1568) at baseline. Stratified analysis was performed based on sex, education, smoking status, and alcohol consumption to examine the longitudinal associations of PEF with the HRs of MCR. In addition, we compared the characteristics of participants with and without PEF. Finally, we reanalyzed the data using a new slower gait cutoff (< 0.654 m/s in men aged < 75 years, < 0.513 m/s in men aged ≥ 75 years, < 0.598 m/s in women aged < 75 years, < 0.424 m/s in women aged ≥ 75 years), which is the highest cutoff of slow gait among seven Health and Retirement Studies (Jayakody et al., 2024).

Stata SE 15.0 (Stata Corp, College Station, TX, USA) was used for analysis. A two-tailed *P* value ≤ 0.05 was considered to indicate statistical significance.

## Results

### Characteristics of the cross-sectional study population

In total, there were 5095 individuals (49.83% female; mean age: 68.74 ± 6.40 years) in the cross-sectional analyses. Individuals with MCR were more likely to have a stroke and lower PEF than those without MCR. The differences in age, sex, education, BMI, smoking status, alcohol consumption status, hypertension status, diabetes status, heart disease status, lung disease status, and asthma status between the two groups were not significant (Table 1).

**TABLE 1:** Baseline characteristics of the cross-sectional study population stratified by the MCR (*n* = 5095).

Characteristics	Total	Without MCR	With MCR	*P*
	*N* = 5095	*N* = 4495	*N* = 600	
Age (years)	68.74 ± 6.40	68.78 ± 6.40	68.43 ± 6.39	0.210
Female	2539 (49.83)	2231 (49.63)	308 (51.33)	0.290
Education level				0.310
Illiterate	1914 (37.57)	1679 (37.35)	235 (39.17)	
Primary school and below	2304 (45.22)	2027 (45.09)	277 (46.17)	
Middle school and above	873 (17.13)	785 (17.46)	88 (14.67)	
BMI	22.88 ± 3.93	22.89 ± 3.93	22.80 ± 3.89	0.570
Smoking status				0.200
Never	2894 (56.80)	2544 (56.60)	350 (58.33)	
Ever smoker	601 (11.80)	532 (11.84)	69 (11.50)	
Current smoker	1568 (30.78)	1387 (30.86)	181 (30.17)	
Alcohol consumption				0.480
Never drinker	2941 (57.72)	2590 (57.62)	351 (58.50)	
Former drinker	588 (11.54)	513 (11.41)	75 (12.50)	
Current drinker	1556 (30.54)	1382 (30.75)	174 (29.00)	
Hypertension	1574 (30.89)	1389 (30.90)	185 (30.83)	0.760
Diabetes	349 (6.85)	307 (6.83)	42 (7.00)	0.930
Heart disease	789 (15.49)	712 (15.84)	77 (12.83)	0.080
Stroke	144 (2.83)	116 (2.58)	28 (4.67)	0.012
Lung disease	710 (13.94)	625 (13.90)	85 (14.17)	0.830
Asthma	1914 (37.57)	1664 (37.02)	250 (41.67)	0.081
PEF (Continuous)	250.37 ± 116.54	252.11 ± 117.07	237.31 ± 111.69	0.003
PEF (Tertiles)				0.010
Lowest	1706 (33.48)	1479 (32.90)	227 (37.83)	
Middle	1634 (32.07)	1437 (31.97)	197 (32.83)	
Highest	1755 (34.45)	1579 (35.13)	176 (29.33)	

The data are presented as the mean ± standard deviation (number, proportion %). Missing data: Education = 4, BMI = 110, smoking status = 32, alcohol consumption = 10, hypertension = 24, diabetes = 40, heart disease = 35, stroke = 17, lung disease = 25, and asthma = 14. PEF, peak expiratory flow; BMI, body mass index.

### Cross-sectional association of PEF with MCR

Multivariate logistic regression analysis revealed that continuous PEF was linked to MCR (OR, 0.998; 95% CI, 0.998, 0.999). The ORs (95% CIs) of incident MCR were 0.857 (0.693, 1.061) and 0.665 (0.524, 0.845) for the middle and highest tertiles of PEF, respectively, compared to the lowest tertile (Table 2).

**TABLE 2:** Odds ratios (ORs) and 95% confidence intervals (CIs) for the cross-sectional association between peak expiratory flow (PEF) and motor cognitive risk syndrome (MCR): results from the logistic regression model.

PEF	Logistic regression model	
	OR (95% CI)^a^	OR (95% CI)^b^
Continuous	0.998 (0.998, 0.999)	0.998 (0.998, 0.999)
**Categorical (tertiles)**		
**Lowest**	**Reference**	**Reference**
Middle	0.871 (0.708, 1.070)	0.857 (0.693, 1.061)
Highest	0.696 (0.554, 0.873)	0.665 (0.524, 0.845)

^a^Adjusted for age, sex, and education. ^b^Adjusted for age, sex, education, body mass index, smoking status, alcohol consumption, hypertension status, diabetes status, stroke status, heart disease status, lung disease status, and asthma status.

### Characteristics of the longitudinal study population

There were 4340 participants (49.52% female; mean age: 68.74 ± 6.41 years) in the longitudinal analyses. Participants in the highest tertile of PEF were more likely to be younger, male, smokers, and drinkers; to have higher education, BMI, and less heart disease and lung disease than those in the lowest tertile (Table 3).

**TABLE 3:** Baseline characteristics of the longitudinal study population by PEF category (*n* = 4340).

Characteristics	PEF (in tertiles)			
	Lowest	Middle	Highest	*P*
	*N* = 1533	*N* = 1435	*N* = 1372	
Age (years)	70.65 ± 6.94	68.52 ± 6.24	66.82 ± 5.26	<0.001
Female	971 (63.34)	814 (56.72)	364 (26.53)	<0.001
Education level				<0.001
Illiterate	767 (50.03)	580 (40.42)	261 (19.02)	
Primary school and below	601 (39.20)	667 (46.48)	715 (52.11)	
Middle school and above	163 (10.63)	187 (13.03)	396 (28.86)	
BMI	22.40 ± 4.05	22.96 ± 3.82	23.33 ± 3.89	<0.001
Smoking status				<0.001
Never	979 (63.86)	886 (61.74)	587 (42.78)	
Ever smoker	166 (10.83)	134 (9.34)	210 (15.31)	
Current smoker	375 (24.46)	405 (28.22)	567 (41.33)	
Alcohol consumption				<0.001
Never drinker	983 (64.12)	893 (62.23)	623 (45.41)	
Former drinker	161 (10.50)	155 (10.80)	173 (12.61)	
Current drinker	386 (25.18)	384 (26.76)	573 (41.76)	
Hypertension	480 (31.31)	432 (30.10)	421 (30.69)	0.830
Diabetes	103 (6.72)	97 (6.76)	100 (7.29)	0.180
Heart disease	289 (18.85)	220 (15.33)	174 (12.68)	<0.001
Stroke	51 (3.33)	38 (2.65)	24 (1.75)	0.087
Lung disease	351 (22.90)	181 (12.61)	83 (6.05)	<0.001
Asthma	595 (38.81)	538 (37.49)	473 (34.48)	0.120

The data are presented as the mean ± standard deviation (number, proportion %). The missing data included BMI = 98, smoking status = 31, alcohol consumption = 9, hypertension = 18, diabetes = 35, heart disease = 32, stroke = 15, lung disease = 22, and asthma = 11. PEF, peak expiratory flow; BMI, body mass index.

### Longitudinal association of PEF with MCR

During the follow-up (accounting for 16782 person-years), a total of 9.88% (429/4340) of participants developed MCR. The proportions of MCR were 12.98% (199/1533), 10.10% (145/1435), and 6.20% (85/1372) for the lowest, middle and highest tertiles of PEF, respectively. According to the multi-adjusted Cox regression models, continuous PEF showed a dose-dependent correlation with the risk of MCR, where each point increase in PEF corresponded to a 2‰ decrease in the risk of MCR. Compared to the lowest tertile of PEF, the HRs (95% CIs) of incident MCR were 0.827 (0.661, 1.036) and 0.576 (0.432, 0.767) for the middle and the highest tertiles, respectively (Table 4).

**TABLE 4:** Hazard ratios (HRs), 95% confidence intervals (CIs) and 10th percentile differences (PDs) in years of incident motor cognitive risk syndrome (MCR) in the longitudinal relation to peak expiratory flow (PEF): results from the Cox regression model and Laplace regression model.

PEF	Cox regression model		Laplace regression model	
	HR (95% CI)^a^	HR (95% CI)^b^	10th PDs (years) (95% CI)^a^	10th PDs (years) (95% CI)^b^
Continuous	0.998 (0.997, 0.999)	0.998 (0.997, 0.999)	0.002 (0.001, 0.003)	0.002 (0.001, 0.003)
**Categorical (Tertiles)**				
**Lowest**	**Reference**	**Reference**	**Reference**	**Reference**
Middle	0.834 (0.671, 1.036)	0.827 (0.661, 1.035)	0.099 (-0.070, 0.267)	0.144 (-0.102, 0.391)
Highest	0.596 (0.452, 0.784)	0.576 (0.432, 0.767)	0.412 (0.118, 0.706)	0.484 (0.151, 0.817)

^a^Adjusted for age, sex, and education. ^b^Adjusted for age, sex, education, body mass index, smoking status, alcohol consumption, hypertension status, diabetes status, stroke status, heart disease status, lung disease status, and asthma status.

Laplace regression analysis showed that the multi-adjusted 10th PDs (95% CI) of time (years) at the onset of MCR for individuals with the highest PEF were 0.484 (0.151, 0.817) years later compared to those with the lowest. Each increase in the PEF score resulted in a 0.002-year delay in the onset of MCR (Table 4).

### Sensitivity analysis

The results remained largely consistent with those of the initial analyses after missing data were imputed by multiple imputations (Supplementary Tables 1, 2) and when 2278 participants with lung disease and asthma at baseline were excluded (Supplementary Tables 3, 4). According to the stratified analyses, the longitudinal associations of PEF with MCR were similar in two subgroups (males and females, illiterate and literate individuals, smokers and nonsmokers, and drinkers and nondrinkers) (Supplementary Tables 5–8). Compared with individuals without PEFs, individuals with PEFs were more likely to be younger, male, smokers, and drinkers; to have a higher education, BMI, and asthma; and to have less hypertension and stroke (Supplementary Table 9). The cross-sectional and longitudinal relationships between PEF and MCR weakened somewhat but remained significant when we used higher cutoff points for slower gait (Supplementary Table 10).

## Discussion

In this nationally representative cohort of older Chinese adults, our findings suggest that higher PEF correlated with a reduced incidence of MCR in a cross-sectional study and a lower risk of MCR in a longitudinal cohort study. Furthermore, the highest tertile of PEF could prolong the onset time of MCR by approximately 0.48 years relative to the lowest tertile.

Pulmonary function may have predictive effects on cognitive performance among middle-aged and older adults (Min et al., 2007; Singh-Manoux et al., 2011; Duggan et al., 2020). However, evidence supporting the longitudinal association of pulmonary function with cognition is scarce and inconsistent. A systematic review (including four longitudinal studies) demonstrated that although substantial cross-sectional studies supported a strong association between pulmonary function and cognition, longitudinal studies supporting these associations were limited (Duggan et al., 2020). Similarly, a cohort study from America suggested that impaired pulmonary function was linked with worse baseline cognitive performance, but no association was detected between lung function and cognitive decline over time (Pathan et al., 2011). One study from the Rush Memory and Aging Project demonstrated that low lung function was related to faster cognitive decline (Wang et al., 2022). Longitudinal data from the CHARLS revealed a significant inverse association between higher baseline PEF and the absolute value of cognitive decline among males but not females (Lai et al., 2022). Another study using the same data also showed that high baseline PEF was independently linked to a slower rate of cognitive decline in older adults in China (Shang et al., 2021).

Furthermore, research on the relationship between lung function and walking speed is relatively rare. A cross-sectional study of the Canadian general population showed that lower lung function was related to perceived poor general health and physical performance (Duong et al., 2022). A study from Sweden indicated that the proportion of individuals with moderate-to-severe walking impairment was markedly greater among individuals with the poorest PEF performance (Trevisan et al., 2020). Data from the Whitehall II study suggested that greater lung function was associated with greater walking speed, memory, and reasoning (Singh-Manoux et al., 2011). However, evidence on the longitudinal association between lung function and walking speed is limited. In addition, MCR, a novel predementia syndrome that combines cognition and physical function indicators, has recently drawn special attention from scholars. To our knowledge, research on the association between lung function and MCR is scarce. An observational study from the National Health and Aging Trends Study revealed that one standard deviation below the mean PEF standardized residual was associated with a 1.60-fold greater odds of developing MCR syndrome (Ho and Verghese, 2023). In our study, using both cross-sectional and longitudinal analyses, we suggested that a higher PEF was associated with a reduced MCR incidence at baseline and was also related to a decreased risk of MCR over time and could further prolong the onset time of MCR, suggesting that early identification of individuals with a changed PEF could facilitate timely interventions to prevent or delay the onset of cognitive decline and mobility impairments. The clinical significance of these findings may provide a new approach to assessing and preventing cognitive risk and offer new ideas for clinical practice and public health policy development.

It is important to discuss the potential influence of the lower gait speed cutoff points used in the CHARLS compared to those used in other studies, such as the U.S. Health and Retirement Study, the English Longitudinal Study of Aging, Survey of Health, Aging and Retirement in Europe, the Harmonized Diagnostic Assessment of Dementia for Longitudinal Aging Study in India, the Mexican Health and Aging Study, and the Brazilian Longitudinal Study of Aging (Jayakody et al., 2024). Considering the racial differences and differences in gait speed measurements, the variability in gait speed has already been identified in previous studies. Thus, the cutoff points of slow gait speed was determined as one standard deviation below the average gait was determined as one standard deviation below the average walking speed for the age- and sex-specific population for each cohort (Oh-Park et al., 2010; Verghese et al., 2013; Cotton et al., 2024). Although the gait speed cutoff points were lower in our study than those in others, the relationship between PEF and MCR was still significant according to the use of higher cutoff points in the sensitivity analyses.

The mechanisms underlying the relationship between PEF and MCR remain unclear. An inflammatory reaction may be initiated by decreased lung function (Reynolds, 1987). This inflammation might progress to low-grade systemic inflammation leading to an adverse vascular reaction and additional oxidative stress (Han et al., 2007). The pathogenesis of dementia is closely linked to inflammation and oxidative stress within the central nervous system (Maccioni et al., 2009). Furthermore, pulmonary dysfunction may also have a direct adverse impact on neurotransmitter synthesis, oxidative stress, and blood-brain barrier integrity via chronic hypoxia and inflammation (Dodd, 2015). Moreover, impaired PEF may lead to clinical complications and reduced physical activity, as well as providing an inadequate peripheral energy supply (Marciniuk, 2008; Roberts and Mapel, 2012).

The strength of the current study is that it is the first study to explore the relationship between lung function and MCR using a representative Chinese cohort of older people. Nevertheless, it is essential to acknowledge several limitations. First, lung function was only assessed by PEF and may not fully reflect the overall lung status. Second, some potential confounders, such as smoking status, education, and chronic diseases, were evaluated through self-reported questions, which may introduce recall bias. Furthermore, although our study fully adjusted for potential sources of bias, the unmeasured confounding factors that may have influenced our findings remained. In addition, the mediation effect of physical activity in the association between PEF and MCR have not found in present study, and this might be due to the missing data on physical activity for 64% of the participants. At present, we have not yet accessed a dataset that includes information on inflammation. Consequently, we are unable to analyze the potential mediating role of inflammation in the association between PEF and MCR. However, we are committed to exploring the mediating effects of inflammation (including a spectrum of inflammation-related indicators) in this association in future research, once the necessary data becomes available. Finally, the generalizability of the findings is restricted considering the individuals with missing PEF data.

In summary, our study presents novel evidence that a high PEF is linked to a decreased prevalence of MCR and may reduce the risk of MCR and further prolong its onset time. Our findings emphasize the significance of maintaining good lung function for physical and cognitive health. Furthermore, additional longitudinal research is necessary to elucidate the mechanisms underlying this relationship in the general population.

## Data availability statement

The datasets presented in this study can be found in online repositories. The names of the repository/repositories and accession number(s) can be found below: http://charls.pku.edu.cn/.

## Ethics statement

The studies involving humans were approved by the Biomedical Ethics Review Committee of Peking University (IRB00001052-11015). The studies were conducted in accordance with the local legislation and institutional requirements. The participants provided their written informed consent to participate in this study.

## Author contributions

HX: Writing–review and editing, Conceptualization. XG: Writing–original draft, Formal analysis, Data curation. KC: Writing–original draft, Formal analysis, Data curation. XL: Writing–review and editing, Formal analysis, Data curation. LC: Formal analysis, Data curation, Writing–review and editing. YiL: Writing–review and editing. YaL: Writing–review and editing. JL: Writing–review and editing, Conceptualization.
