# Supplementary material for: Association of peak expiratory flow with motoric cognitive risk syndrome among older adults
Source: Front Aging Neurosci. 2024 Aug 7;16:1412542. doi: 10.3389/fnagi.2024.1412542 (PMC11335682; doi:10.3389/fnagi.2024.1412542)
Supplement: Supplementary file 1 [file Table_1.docx]

**Supplementary Table 1.** Odds ratio (ORs) and 95% CIs (confidence intervals) for the cross-sectional association between peak expiratory flow (PEF) and motor cognitive risk syndrome (MCR) with multiple imputation for missing data: results from Logistic regression model.

| **PEF** | **Logistic regression model** | |
| --- | --- | --- |
|  | OR (95% CI)^a^ | OR (95% CI)^b^ |
| Continuous | 0.999 (0.998, 0.999) | 0.999 (0.998, 0.999) |
| Categorical (tertiles) |  |  |
| Lowest | Reference | Reference |
| Middle | 0.871 (0.708, 1.070) | 0.866 (0.703, 1.068) |
| Highest | 0.696 (0.554, 0.873) | 0.686 (0.543, 0.869) |

^a^ Adjusted for age, sex, and education.

^b^ Adjusted for age, sex, education, body mass index, smoking, alcohol consumption, hypertension, diabetes, stroke, heart disease, lung disease, and asthma.

**Supplementary Table 2.** Harzads ratios (HRs) and 95% CIs (confidence intervals) and 10th percentile differences (PDs) in years of incident motor cognitive risk syndrome (MCR) in relation to peak expiratory flow (PEF) with multiple imputation for missing data: results from Cox regression model and Laplace regression model.

| **PEF** | **Cox regression model** | |  | **Laplace regression model** | |
| --- | --- | --- | --- | --- | --- |
|  | HR (95% CI)^a^ | HR (95% CI)^b^ |  | 10th PDs (years) (95% CI)^a^ | 10th PDs (years) (95% CI)^b^ |
| Continuous | 0.998 (0.997, 0.999) | 0.998 (0.997, 0.999) |  | 0.002 (0.001, 0.003) | 0.002 (0.001, 0.003) |
| Categorical (tertiles) |  |  |  |  |  |
| Lowest | Reference | Reference |  | Reference | Reference |
| Middle | 0.834 (0.671, 1.036) | 0.836 (0.671, 1.042) |  | 0.101 (-0.073, 0.274) | 0.138 (-0.066, 0.342) |
| Highest | 0.596 (0.453, 0.785) | 0.597 (0.450, 0.793) |  | 0.415 (0.119, 0.712) | 0.458 (0.118, 0.797) |

^a^ Adjusted for age, sex, and education.

^b^ Adjusted for age, sex, education, body mass index, smoking, alcohol consumption, hypertension, diabetes, stroke, heart disease, lung disease, and asthma.

**Supplementary Table 3.** Odds ratio (ORs) and 95% CIs (confidence intervals) for the association between peak expiratory flow (PEF) and motor cognitive risk syndrome (MCR) when participants with lung disease and asthma at baseline were excluded: results from Logistic regression model.

| **PEF** | **Logistic regression model** | |
| --- | --- | --- |
|  | OR (95% CI)^a^ | OR (95% CI)^b^ |
| Continuous | 0.998 (0.997, 0.999) | 0.998 (0.997, 0.999) |
| Categorical (tertiles) |  |  |
| Lowest | Reference | Reference |
| Middle | 0.713 (0.535, 0.950) | 0.690 (0.514, 0.927) |
| Highest | 0.578 (0.416, 0.802) | 0.581 (0.416, 0.813) |

^a^ Adjusted for age, sex, and education.

^b^ Adjusted for age, sex, education, body mass index, smoking, alcohol consumption, hypertension, diabetes, stroke, and heart disease.

**Supplementary Table 4.** Harzads ratios (HRs) and 95% CIs (confidence intervals) and 10th percentile differences (PDs) in years of incident motor cognitive risk syndrome (MCR) in relation to peak expiratory flow (PEF) when participants with lung disease and asthma at baseline were excluded: results from Cox regression model and Laplace regression model.

| **PEF** | **Cox regression model** | |  | **Laplace regression model** | |
| --- | --- | --- | --- | --- | --- |
|  | HR (95% CI)^a^ | HR (95% CI)^b^ |  | 10th PDs (years) (95% CI)^a^ | 10th PDs (years) (95% CI)^b^ |
| Continuous | 0.998 (0.997, 0.999) | 0.998 (0.997, 0.999) |  | 0.001 (0.001, 0.003) | 0.002 (0.001, 0.004) |
| Categorical (tertiles) |  |  |  |  |  |
| Lowest | Reference | Reference |  | Reference | Reference |
| Middle | 0.856 (0.641, 1.143) | 0.837 (0.622, 1.126) |  | 0.082 (-0.132, 0.297) | 0.186 (-0.200, 0.572) |
| Highest | 0.577 (0.398, 0.836) | 0.548 (0.375, 0.800) |  | 0.449 (0.061, 0.838) | 0.557 (0.032, 1.146) |

^a^ Adjusted for age, sex, and education.

^b^ Adjusted for age, sex, education, body mass index, smoking, alcohol consumption, hypertension, diabetes, stroke, heart disease, lung disease, and asthma.

**Supplementary Table 5.** Harzads ratios (HRs) and 95% CIs (confidence intervals) of motor cognitive risk syndrome (MCR) in relation to peak expiratory flow (PEF) by sex.

| **Gender** | **PEF** | **Cox regression model** |
| --- | --- | --- |
|  |  | HR (95% CI)^b^ |
| Male | Continuous | 0.998 (0.997, 0.999) |
|  | Categorical (tertiles) |  |
|  | Lowest | Reference |
|  | Middle | 0.788 (0.545, 1.138) |
|  | Highest | 0.553 (0.374, 0.817) |
| Female | Continuous | 0.998 (0.996, 0.999) |
|  | Categorical (tertiles) |  |
|  | Lowest | Reference |
|  | Middle | 0.849 (0.639, 1.128) |
|  | Highest | 0.587 (0.373, 0.925) |

^a^ Adjusted for age, education, body mass index, smoking, alcohol consumption, hypertension, diabetes, stroke, and heart disease.

**Supplementary Table 6.** Harzads ratios (HRs) and 95% CIs (confidence intervals) of motor cognitive risk syndrome (MCR) in relation to peak expiratory flow (PEF) by education.

| **Education** | **PEF** | **Cox regression model** |
| --- | --- | --- |
|  |  | HR (95% CI)^b^ |
| Illiterate | Continuous | 0.998 (0.996, 0.999) |
|  | Categorical (tertiles) |  |
|  | Lowest | Reference |
|  | Middle | 0.808 (0.591, 1.106) |
|  | Highest | 0.522 (0.316, 0.864) |
| Literate | Continuous | 0.998 (0.997, 0.999) |
|  | Categorical (tertiles) |  |
|  | Lowest | Reference |
|  | Middle | 0.858 (0.619, 1.188) |
|  | Highest | 0.581 (0.404, 0.837) |

^a^ Adjusted for age, education, body mass index, smoking, alcohol consumption, hypertension, diabetes, stroke, and heart disease.

**Supplementary Table 7.** Harzads ratios (HRs) and 95% CIs (confidence intervals) of motor cognitive risk syndrome (MCR) in relation to peak expiratory flow (PEF) by smoking.

| **Smoking** | **PEF** | **Cox regression model** |
| --- | --- | --- |
|  |  | HR (95% CI)^b^ |
| Never or ever | Continuous | 0.998 (0.996, 0.999) |
|  | Categorical (tertiles) |  |
|  | Lowest | Reference |
|  | Middle | 0.748 (0.575, 0.973) |
|  | Highest | 0.557 (0.392, 0.790) |
| Current | Continuous | 0.998 (0.997, 0.999) |
|  | Categorical (tertiles) |  |
|  | Lowest | Reference |
|  | Middle | 0.986 (0.624, 1.560) |
|  | Highest | 0.647 (0.384, 0.815) |

^a^ Adjusted for age, education, body mass index, smoking, alcohol consumption, hypertension, diabetes, stroke, and heart disease.

**Supplementary Table 8.** Harzads ratios (HRs) and 95% CIs (confidence intervals) of motor cognitive risk syndrome (MCR) in relation to peak expiratory flow (PEF) by alcohol consumption.

| **Alcohol** | **PEF** | **Cox regression model** |
| --- | --- | --- |
|  |  | HR (95% CI)^b^ |
| Never or ever | Continuous | 0.998 (0.997, 0.999) |
|  | Categorical (tertiles) |  |
|  | Lowest | Reference |
|  | Middle | 0.826 (0.638, 1.069) |
|  | Highest | 0.657 (0.467, 0.925) |
| Current | Continuous | 0.997 (0.995, 0.999) |
|  | Categorical (tertiles) |  |
|  | Lowest | Reference |
|  | Middle | 0.838 (0.528, 1.330) |
|  | Highest | 0.460 (0.271, 0.781) |

^a^ Adjusted for age, education, body mass index, smoking, alcohol consumption, hypertension, diabetes, stroke, and heart disease.

**Supplementary Table 9.** The characteristics of the study population with and without PEF.

| **Characteristics** | Total | Without PEF | With PEF | *P* |
| --- | --- | --- | --- | --- |
|  | N=6690 | N=1584 | N=5106 |  |
| Age (years) | 69.16±6.82 | 70.49±7.87 | 68.75±6.41 | <0.001 |
| Female | 3,327 (49.73) | 783 (49.43) | 2,544 (49.82) | <0.001 |
| Education level |  |  |  | <0.001 |
| Illiterate | 2,497 (37.32) | 577 (36.43) | 1,920 (37.60) |  |
| Primary school and below | 2,901 (43.36) | 593 (37.44) | 2,308 (45.20) |  |
| Middle school and above | 1,250 (18.68) | 377 (23.80) | 873 (17.10) |  |
| BMI | 22.84±3.92 | 22.03±3.72 | 22.88±3.92 | <0.001 |
| Smoking status |  |  |  | <0.001 |
| Never | 3,830 (57.25) | 929 (58.65) | 2,901 (56.82) |  |
| Ever smoker | 741 (11.08) | 140 ( 8.84) | 601 (11.77) |  |
| Current smoker | 1,882 (28.13) | 314 (19.82) | 1,568 (30.71) |  |
| Alcohol consumption |  |  |  | <0.001 |
| Never drinker | 3,889 (58.13) | 941 (59.41) | 2,948 (57.74) |  |
| Former drinker | 746 (11.15) | 158 ( 9.97) | 588 (11.52) |  |
| Current drinker | 1,970 (29.45) | 411 (25.95) | 1,559 (30.53) |  |
| Hypertension | 2,098 (31.36) | 522 (32.95) | 1,576 (30.87) | <0.001 |
| Diabetes | 448 ( 6.70) | 98 ( 6.19) | 350 ( 6.85) | <0.001 |
| Heart disease | 1,040 (15.55) | 250 (15.78) | 790 (15.47) | <0.001 |
| Stroke | 205 ( 3.06) | 60 ( 3.79) | 145 ( 2.84) | <0.001 |
| Lung disease | 923 (13.80) | 210 (13.26) | 713 (13.96) | <0.001 |
| Asthma | 2,395 (35.80) | 478 (30.18) | 1,917 (37.54) | <0.001 |

Data are presented as mean ± standard deviations, number (proportion %).

Missing data: Education=42, BMI=142, Smoking status=237, Alcohol consumption=85, Hypertension=98, Diabetes=122, Heart disease=110, Stroke=88, Lung disease=98, Asthma=86. Abbreviations: PEF, peak expiratory flow; BMI, Body mass index.

**Supplementary Table 10.** The cross-sectional and longitudinal relationships between peak expiratory flow (PEF) and motor cognitive risk syndrome (MCR) in the Chinese older adults with a new slower gait speed cutoff.

| **PEF** | **Logistic regression model** | | | |  | **Cox regression model** | | | |
| --- | --- | --- | --- | --- | --- | --- | --- | --- | --- |
|  | n | case | OR (95% CI)^a^ | OR (95% CI)^b^ |  | n | case | HR (95% CI)^a^ | HR (95% CI)^b^ |
| Continuous | 5077 | 1589 | 0.999 (0.998, 0.999) | 0.999 (0.998, 0.999) |  | 3365 | 859 | 0.999 (0.998, 0.999) | 0.998 (0.998, 0.999) |
| Categorical (tertiles) |  |  |  |  |  |  |  |  |  |
| Lowest | 1698 | 563 | Reference | Reference |  | 1169 | 343 | Reference | Reference |
| Middle | 1629 | 530 | 0.922 (0.795, 1.068) | 0.911 (0.782, 1.061) |  | 1108 | 300 | 0.905 (0.774, 1.059) | 0.907 (0.771, 1.068) |
| Highest | 1750 | 496 | 0.708 (0.604, 0.831) | 0.689 (0.583, 0.815) |  | 1088 | 216 | 0.679 (0.563, 0.818) | 0.67 9(0.558, 0.825) |

^a^ Adjusted for age, sex, and education.

^b^ Adjusted for age, sex, education, body mass index, smoking, alcohol consumption, hypertension, diabetes, stroke, heart disease, lung disease, and asthma.
